# A Single-Point Insulin Sensitivity Estimator (SPISE) of 5.4 is a good predictor of both metabolic syndrome and insulin resistance in adolescents with obesity

**DOI:** 10.3389/fendo.2023.1078949

**Published:** 2023-02-08

**Authors:** Paulina Correa-Burrows, Mariela Matamoros, Valeria de Toro, Diego Zepeda, Marta Arriaza, Raquel Burrows

**Affiliations:** ^1^ Instituto de Nutrición y Tecnología de los Alimentos, Universidad de Chile, Santiago, Chile; ^2^ Departamento de Gastroenterología y Nutrición Pediátrica, Pontificia Universidad Católica de Chile, Santiago, Chile; ^3^ Instituto de Investigación Materno Infantil, Facultad de Medicina, Universidad de Chile, Santiago, Chile; ^4^ Servicio de Pediatría, Hospital Gustavo Fricke, Viña del Mar, Chile; ^5^ Programa Clínico de Obesidad Infantil (POI), Instituto de Nutrición y Tecnología de los Alimentos, Universidad de Chile, Santiago, Chile

**Keywords:** Single-Point Insulin Sensitivity Estimator, pediatric obesity, insulin resistance, metabolic syndrome, cardiometabolic risk

## Abstract

**Background:**

The Single-Point Insulin Sensitivity Estimator (SPISE) is a biomarker of insulin sensitivity estimated using BMI and triglycerides and high-density lipoprotein cholesterol. We assessed the accuracy of SPISE to screen obesity-related cardiometabolic risk in children and adolescents.

**Method:**

Cross-sectional validation study for a screening test in a sample of *n*=725 children and adolescents from an obesity clinic. Weight, height, waist circumference, blood arterial pressure, lipid profile, glucose, insulin and Tanner stage were measured. BMI, BMI for-age-and sex (BAZ), and HOMA-IR were estimated. HOMA-IR values ≥2.1 and ≥3.3 were considered IR in Tanner I-II, ≥3.3 for Tanner III-IV and ≥2.6 for Tanner V, respectively. Metabolic Syndrome (MetS) was diagnosed with the Cook phenotype. SPISE was estimated according to the following algorithm: [600* HDL^0.185/(TG^0.2* BMI^1.338)]. The optimal SPISE cut points for IR and MetS prediction were determined by ROC curve analysis.

**Results:**

In prepubertal obese patients (9.2 ± 2.1y; 18.4% males), the prevalence of IR and MetS was 28.2% y 46.9%, respectively; 58% had severe obesity (BAZ ≥4 *SD*). In pubertal obese patients (12.6 ± 1.8y; 57% males), the prevalence of IR and MetS was 34.1% and 55.3%, respectively; 34% had severe obesity. In prepubertal children, a SPISE of 6.3 showed the highest sensitivity (73.2%) and specificity (80%) to screen individuals with IR (AUC: 0.80; LR +: 3.3). Likewise, a SPISE of 5.7 got the highest sensitivity (82.6%) and specificity (86.1%) to screen patients with MetS (AUC: 0.87; LR +: 5.4). In pubertal patients, a SPISE of 5.4 showed the highest sensitivity and specificity to screen children and adolescents with both IR (Sn: 76.1%; Sp: 77.5%; AUC: 0.8; LR +: 3.1) and MetS (Sn: 90.4%; Sp: 76.1%; AUC: 0.90; LR +: 3.5).

**Conclusion:**

In children and adolescents with obesity, SPISE has good or very good performance in predicting IR and MetS. SPISE may be considered a relatively simple and low-cost diagnosis tool that can be helpful to identify patients with greater biological risk. In adolescents with obesity, the same cut point allows identification of those at higher risk of both IR and MetS.

## Introduction

Obesity increases the risk of cardiometabolic diseases, musculoskeletal disorders, and certain types of cancers (1). Once considered a high-income nation problem, it is now on the rise also in non-industrialized countries (2–4). Worldwide, the prevalence in adults nearly tripled since the 1970s, while in children and adolescents rose tenfold (5, 6). Obesity has reached pandemic proportions as no country has successfully reduced rates through the years. By 2025, the prevalence will reach 18% and 21% in males and females, respectively (5). The prevalence of obesity is high in Chile, with ~35% of the population >15y having the condition. This rate is higher in socially vulnerable groups: 47% (7). Despite persistent efforts to tackle childhood obesity,31% of school-age children have obesity and 27% have overweight (8).

Complications of childhood obesity, including as insulin resistance (IR) and metabolic syndrome (MetS), explain the onset of type-2 diabetes (T2DM) and cardiovascular diseases at increasingly younger ages (9–11). Evidence shows a high prevalence of IR and MetS in the population with obesity and below the age of 16 years (12–15). Yet, the reported rates vary considerably due to a lack of consensus on diagnosing these disorders, which also difficult their clinical management (16, 17). Although impaired insulin sensitivity and altered pancreatic β-cell functioning are crucial in the pathogenesis of MetS and T2DM, diagnosis of IR based on fasting insulin measurement is not recommended (18, 19). Insulin determination involves costly procedures, complex methodologies, and elaborated sample management and storage (13, 18, 19). Also, pulsatile insulin secretion is considered physiological; hence, substantial variations may exist in the plasmatic secretion of this hormone, which requires several measurements to obtain reliable values (20, 21).

The Single-Point Insulin Sensitivity Estimator (SPISE) is a relatively simple mathematical method for estimating insulin sensitivity based on routine clinical measurements and labs such as BMI, triglycerides (TG), and HDL cholesterol (22). When screening IR in obese Caucasian adolescents, the performance of SPISE was only exceeded by the Matsuda Index, although the algorithm does not include insulin and glycemia values. Likewise, SPISE had a better predictive performance of IR than the Homeostatic Model Assessment (HOMA-IR) and the Quantitative Insulin Sensitivity Check Index (QUICKI) (22). The authors found the same performance pattern in the screening of IR in Caucasian adults with obesity (22). Subsequently, SPISE showed good clinical performance in diagnosing MetS, T2DM, and coronary heart disease in adults and the elderly (23, 24).

So far, very few studies assessed SPISE performance for abnormal glucose metabolism or IR-related cardiometabolic disorders diagnosis in younger-age people (22, 25, 26); the majority was conducted in adolescents with complete pubertal development. In puberty, however, considerable metabolic and hormonal change occur resulting in a marked decrease in insulin sensitivity (IS). A nadir in IS occurs around mid-puberty in healthy subject, which should recover at puberty completion (27, 28). Failure to solve IR in children with obesity going into puberty may result in greater cardiometabolic risk.

The validity assessment of SPISE for screening of IR-related cardiometabolic risk is in great need in the pediatric population exposed to overnutrition and at different degrees of biological maturity. Consequently, this study aims to determine the clinical and analytic performance of this algorithm for IR and MetS screening in Chilean pre-pubertal and pubertal patients from an obesity clinic based in Santiago. Also, we will estimate optimal cutoffs for MetS and IR prediction according to developmental stage.

## Methods

### Participants and study design

Cross-sectional validation study for a screening test. Between 2006 and 2016, n=850 obese 5 to 15-year-olds were evaluated at the Pediatric Clinical Obesity Program, Institute of Nutrition and Food Technology, Universidad de Chile. A total of n=725 had completed laboratory assessments and, thus, were considered for the analysis. The Scientific Ethics Committee of the Institute of Nutrition and Food Technology (INTA) at the University of Chile granted approval for this study, according to the norms for Human Experimentation, Code of Ethics of the World Medical Association. Parents or primary caregivers signed informed consent and participants assented.

### Measurements

#### Anthropometric and pubertal assessment

A trained pediatric endocrinologist measure weight (kg) to the nearest 0.1 kg, using a scale (703, Seca GmbH & co. Hamburg, Germany), and height (cm) to the nearest 0.1 cm, using a Holtain stadiometer. Waist circumference (WC) was measured to the nearest millimeter at the level of the umbilicus, using automatic-fixing flexible tape measures (Seca 201, Seca GmbH & co. Hamburg, Germany). Measurements were taken twice, with a third measurement, if necessary. BMI and BMI-for-age-and-sex (BAZ) were computed. The 2007 WHO references were used for obesity diagnosis (BAZ ≥2 *SD*) (29). WC percentiles were calculated as indicated on the US Centers for Disease Control and Prevention Growth Charts (30). Pubertal development was assessed from physical examination of the patient, according to the Marshall & Tanner criteria for breast and genital stage in females and males, respectively (31, 32). Tanner stages I-II were categorized as pre-pubertal or children, and Tanner stages III-IV were categorized as pubertal or adolescents.

#### Cardiometabolic risk assessment

Blood Pressure was measured and evaluated following the standard recommended by the National High Blood Pressure Education Working Group (33). Fasting venous blood samples were obtained after 8-12-h overnight fast. Serum glucose concentration was determined through the colorimetric enzymatic method (Química Clínica Aplicada, S.A, Amposta, Spain). Insulin was determined by radioimmunoassay (Diagnostic Products Corporation, Los Angeles, CA, intra-assay variation ≤5.1%, interassay variation ≤7.1%). Cholesterol profile was determined by dry analytical methodology (Roche Diagnostics, Mannheim, Germany). TG | HDL-chol ratio was computed, and LDL was estimated using the Friedewald equation: [TChol - (HDL-chol + TG/5)]. HOMA was used to measure IR and β-cell function. HOMA-insulin resistance (IR) was estimated as the product of fasting glucose (mmol/l) and insulin (μU/ml) divided by 22.5. IR was diagnosed according to national references: HOMA-IR ≥2.1 for Tanner I and II; HOMA-IR ≥3.3 for Tanner III and IV, HOMA-IR ≥2.6 for Tanner V (34, 35). For HOMA-β the following formula was used: HOMA-β = [20 × Ins(µU/l)]/[(Glu(mg/dl) × 0.0555)–3.5], whereas HOMA-S was calculated as: HOMA-S = [1/HOMA-IR] × 100%]. Disposition Index (DI), also a marker of β-cell functioning, was computed as DI=[(HOMA-S1/100) × (HOMA-β1/100)]. SPISE was calculated according to algorithm proposed by Paulmichl et al: [600 * HDL^0.185/(TG^0.2 * BMI^1.338)] (22). A continuous value was obtained, ranging 2.5-14.5 with higher values denoting higher insulin sensitivity. The Metabolic Syndrome (MetS) was diagnosed with the Cook et al. standard (36).

### Data analysis

The normality assumption was tested (Shapiro-Wilk) in all variables. The Student’s *t*-test for and Mann–Whitney U test were used for comparison of mean or median values of anthropometric and cardiometabolic markers. The Pearson’s chi-squared test was used for comparison of categorical variables and determine statistical independence. Receiver operating characteristic (ROC) analysis was used to find the optimal cutoff of SPISE for MetS and IR screening in children and adolescents. With this methods, sensitivity (Sn), specificity (Sp), likelihood ratio (LR), and area under the ROC curve (AUC) were computed. The Youden Index [*J*=sensitivity-(1-specificity)] was used to determine the optimal cutoffs for IR and MetS prediction. Then, the values were verified with the LR for a positive result. The proportion of participants below cutoffs who truly have the MetS or IR, also known as post-test probability, was estimated (Fagan’s nomogram). To compare the screening performance of SPISE in the prediction of MetS and IR in pubertal vs. prepubertal patients, we used the DeLong’s method for pair design, which allow testing the statistical significance of the difference between the AUC. Last, we examined whether our SPISE cutoffs for MetS and IR screening were associated with greater cardiometabolic risk in the group having a SPISE below those cutoffs. Cohen’s *d* and Cliff’s δ were used to indicating the standardized difference between mean and median values, respectively. Values of *d* of 0.20, 0.50 and 0.80 denote small, medium and large differences between means (37), whereas the absolute value of δ can be considered small around 0.15, medium around 0.33, and large around 0.47 (38). Stata for Windows V.15.0 (Lakeway Drive College Station, Texas, USA) was used for data processing.

## Results

The clinical sample of n=725 obese Chilean children and adolescents is described in **Table 1**. The results are presented by pubertal stage. In prepubertal obese patients (9.1 ± 2.0y; 82% females), the prevalence of IR and MetS was 47% y 28%, respectively; 58% had severe obesity (BAZ ≥ 4 *SD*). In pubertal obese patients (12.6 ± 1.8; 43% females), the prevalence of IR and MetS was 55% and 34%, respectively; 34% had severe obesity. The prevalence of abdominal obesity was significantly higher in children, however, the prevalence of hypertriglyceridemia and low-HDL chol was higher in the adolescent group. Other cardiometabolic risk factors showed no difference between the groups. Biomarkers of β-cell functioning, such as HOMA-IR, HOMA-S, and HOMA-β, were much deteriorated in the pubertal category. While DI showed no difference in children *vs* adolescents, it is worth mentioning that in both this ratio was well below 1.0, denoting a reduced β-cell ability to compensate for insulin resistance. Values of SPISE were much lower in the adolescent group compared to children, denoting worse insulin sensitivity.

**Table 1:** Anthropometric characteristics and cardiometabolic profile in children and adolescents with obesity (n=725).

Variable	Children (Tanner 1-2) (n=432)	Adolescents (Tanner 3-5) (n=293)	*P* value
Age (years)	9.2 ± 2.1	12.6 ± 1.8	<0.001
Sex (females)	81.6%	43.1%	<0.001
Body-Mass Index (z score)	4.50 ± 1.8	3.75 ± 1.7	<0.001
Severe obesity (%)	57.9%	33.5%	<0.001^ǂ^
Height (z score)	0.53 ± 0.9	0.40 ± 1.0	NS
Waist circumference (cm)	83.5 ± 10.5	93.8 ± 11.7	<0.001
Systolic Blood Pressure (mm Hg)	105 ± 12	114 ± 14	<0.001
Diastolic Blood Pressure (mm Hg)	66 ± 9	72 ± 9	<0.001
Fasting glucose (mg/dl)	86.5 ± 9.3	86.9 ± 9.3	NS
Fasting insulin (uUI/dl)	9.0 (10.0)	14.4 (11.3)	<0.001^§^
HOMA-IR	1.93 (2.1)	3.04 (2.5)	<0.001^§^
HOMA-S (%)	55.5 (60)	34.5 (27)	<0.001^§^
HOMA-β (%)	142.2 (155)	240.8 (236)	<0.001^§^
Disposition Index	0.73 (0.4)	0.75 (0.5)	NS
Total cholesterol (mg/dl)	171.7 ± 32.9	166.1 ± 35.2	0.046
LDL cholesterol (mg/dl)	104.2 ± 29.6	99.7 ± 30.7	0.023
HDL cholesterol (mg/dl)	47.5 ± 10.2	44.9 ± 9.8	<0.001
Triglycerides (mg/dl)	85.4 ± 61.0	100.0 ± 72.9	0.002^§^
TG-HDL ratio	2.38 ± 1.8	2.68 ± 1.8	0.036
SPISE Index	6.81 ± 1.6	5.71 ± 1.5	<0.001
*Cardiometabolic risk factors*			
Abdominal obesity (%)	92.6%	87.7%	0.027^ǂ^
High Blood Pressure	36.3%	40.6%	NS^ǂ^
Hyperglycemia	7.9%	8.8%	NS^ǂ^
Hypertriglyceridemia	33.6%	41.6%	0.027^ǂ^
Low HDL-Cholesterol	26.9%	36.5%	0.006^ǂ^
Metabolic Syndrome (%)	28.2%	34.1%	*0.091^ʃ^*
Insulin resistance (%)	46.9%	55.3%	0.026^ǂ^

Values are Mean ± SD, Median (IQR), and relative frequencies. Two-tailed Student’s t test for independent samples, except as indicated. ^§^Wilcoxson rank-sum test. ^ǂ^ χ^2^ (Pearson). ^ʃ^Trend towards significance. MetS and its components were diagnosed according to the Cook standard. Insulin resistance was diagnosed with HOMA-IR values ≥2.1 (Tanner 1-2), ≥3.3 (Tanner 3-4), ≥2.6 (Tanner 5). Severe obesity: BMI for-age-and-sex ≥ 4 SD. ^ʃ^Trend towards significance.

In prepubertal children, a SPISE of 6.3 showed the highest sensitivity (73.2%) and specificity (80%) to identify individuals with IR (AUC: 0.80; LR +: 3.3) (**Table 2**). Likewise, a SPISE of 5.7 had the highest sensitivity (82.6%) and specificity (86.1%) to screen MetS (AUC: 0.87; LR +: 5.4). In pubertal patients, a SPISE of 5.4 showed the highest sensitivity and specificity to screen both IR (Sn: 76.1%; Sp: 77.5%; AUC: 0.8; LR +: 3.1) and MetS (Sn: 90.4%; Sp: 76.1%; AUC: 0.90; LR +: 3.5).

**Table 2:** Optimal cutoff values and summary indices of SPISE performance to predict Insulin Resistance and Metabolic Syndrome in children and adolescents with obesity (n=725).

	Children (Tanner 1-2) (n=432)		Adolescents (Tanner 3-5) (n=293)	
	Metabolic Syndrome	Insulin Resistance	Metabolic Syndrome	Insulin Resistance
Optimal cutoff	5.7	6.3	5.4	5.4
Sensitivity (%)	82.6	73.2	90.4	76.1
Specificity (%)	86.1	80.0	76.1	77.6
Correctly Classified (%)	83.8	75.2	79.0	74.5
Positive Predicted Value	61.9	72.1	64.5	73.9
Negative Predicted Value	94.7	80.7	94.4	79.4
False Positive Fraction	18.5	26.2	31.3	30.7
False Negative Fraction	27.4	34.7	17.2	32.9
Positive likelihood ratio	5.4	3.3	3.3	3.1
Negative likelihood ratio	0.51	0.61	0.45	0.66
Pre-test probability (prevalence)	21.5	41.9	31.2	45.7
Post-test probability (having a test positive)	64%-72%	52%-62%	65%-74%	55%-66%
Area Under Curve	0.873	0.795	0.895	0.800

The chance that MetS or IR will be present when a positive test result was obtained by calculating the post-test probability. For ease of interpretation, **Table 2** contains the probabilities of the presence of the MetS and IR before and after using the SPISE. In prepubertal patients, the pretest probability of having the MetS was 21.5% before the SPISE. After the SPISE, for those with values below 5.7 (positive test), the chances of having the disease increased to a range of 64%-72%. In the same group, the pretest probability of having IR was 41.9% before the SPISE. After the SPISE, for those having values below 6.4, the chances of having the disease increased to a range of 52%-62%. The same pattern was found in the pubertal group, for whom the probability of having MetS or IR increased notably in those with a SPISE below 5.4.

**Figure 1:**
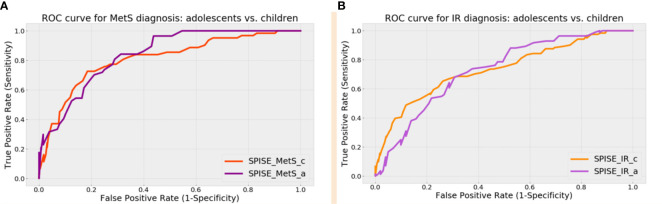
ROC Curves for MetS **(A)** and IR **(B)** screening: children vs. adolescents.

Next, we compared the screening performance of SPISE for MetS and IR prediction in pre- pubertal vs. pubertal patients (**Figure 1** and **Table 3**). Although AUCs were slightly superior in the pubertal group for both MetS and IR screening, the DeLong test showed that the difference in the areas were not statistically significant. This suggests that SPISE works equally well in identifying subjects with these two cardiometabolic conditions despite their developmental stage.

**Table 3:** ROC Curves comparison for MetS and IR screening: children vs. Adolescents.

Screening performance for MetS		Screening performance for IR		
AUC SPISE_MetS children	0.873	AUC SPISE_IR children	0.795
AUC SPISE_MetS adolescents	0.895	AUC SPISE_IR adolescents	0.800
Difference: SPISE_CHLD_ - SPISE_ADOL_	0.022	Difference: SPISE_CHLD_ - SPISE_ADOL_	0.005
Standard error of the difference	0.036	Standard error of the difference	0.042
Z statistic	0.620	Z statistic	0.116
*P* value (two-tailed)	0.535	*P* value (two-tailed)	0.907

**Figure 2:**
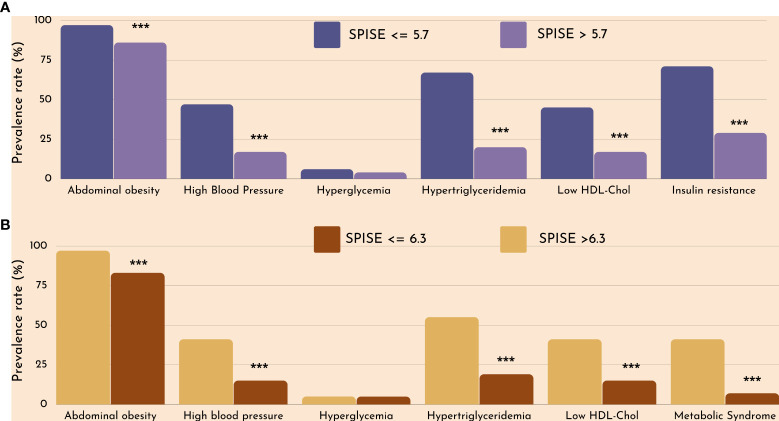
Cardiometabolic risk in obese children according to optimal SPISE cutoffs for MetS **(A)** and IR **(B)** screening. MetS and components diagnosed with the Cook et al. phenotype. Insulin Resistance defined as having HOMA-IR ≥2.1 (Tanner 1-2). Pearson’s χ2 test: ^***^Significance at α<0.0001.

**Figure 3:**
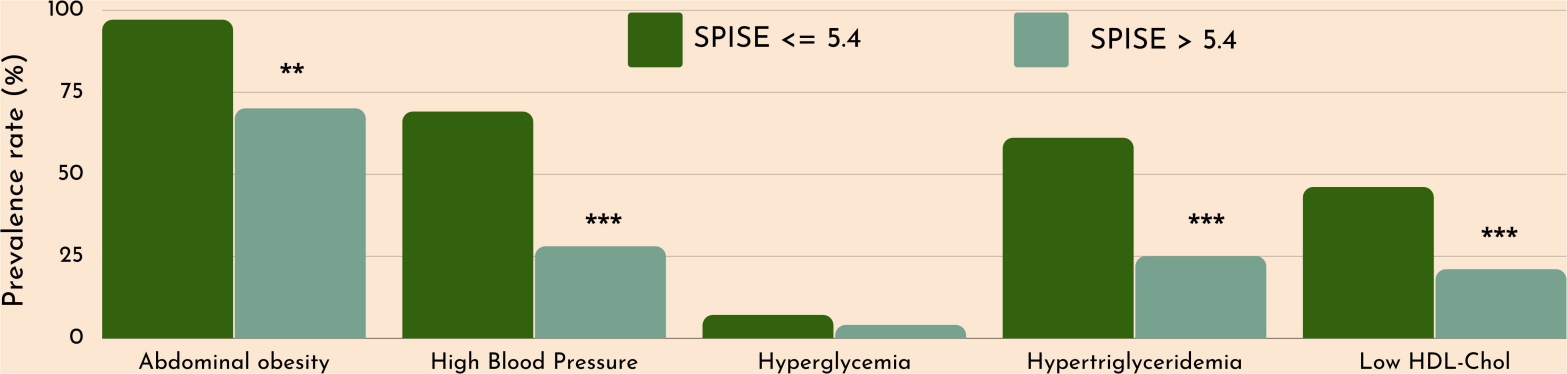
Cardiometabolic risk in obese adolescents according to optimal SPISE cutoffs for MetS and IR screening (SPISE ≤5.4). MetS components diagnosed with the Cook et al. phenotype. Pearson’s χ^2^ test: *Significance at α=0.05. ***Significance at α<0.0001.

Finally, we checked whether our SPISE cutoffs related to higher biological risk in patients with a SPISE below the cutoffs for prediction of MetS and IR. In both prepubertal and pubertal patients having the test positive, we found a higher prevalence of hypertriglyceridemia and low HDL. However, we also observed a higher prevalence of high blood pressure and abdominal obesity, whose biomarkers are not nor part of the SPISE algorithm (**Figures 2**, **3**). **Table 4** reports the cardiometabolic profile of children or prepubertal patients after controlling for MetS and IR presence, according to SPISE cutoffs. Children having SPISE values below the cutoff for MetS (5.7) and IR (6.3) prediction had significantly higher values of WC, SBP, DBP, insulin, HOMA-IR, HOMA-s, HOMA-β, TChol, TG/HDL-chol ratio and TG, and significantly lower values of HDL-chol compared to those having the test negative. The effect size (ES) for difference was also large for those biomarkers not included in the SPISE algorithm (WC, insulin), as well as for surrogates to quantify IR and β-cell function (HOMA-IR, HOMA-S, and HOMA-β). The same pattern was found in adolescent patients, as shown in **Table 5**. Again, the effect size (ES) for difference was large for WC, insulin, HOMA-IR and HOMA-S.

**Table 4:** Cardiometabolic profile in prepubertal obese patients according to optimal cutoff values of SPISE to predict IR (6.3) and MetS (5.7).

	Insulin Resistance				Metabolic Syndrome			
Variable	SPISE ≤ 6.3 (n=173)	SPISE > 6.3 (n=259)	Mean diff	ES for diff^†^	SPISE ≤ 5.7 (n=118)	SPISE > 5.7 (n=314)	Mean diff	ES for diff^†^
Waist circumference (mm Hg)	91.0 ± 9.0^***^	78.6 ± 7.5	12.4	1.52	93.6 ± 9.4^***^	79.8 ± 8.0	13.8	1.40
Systolic Blood Pressure (mm Hg)	109 ± 13^***^	101 ± 10	8.0	0.71	109 ± 13^***^	102 ± 10	7.6	0.64
Diastolic Blood Pressure (mm Hg)	69 ± 9^***^	65 ± 8	4.0	0.48	69 ± 9^***^	64 ± 8	4.7	0.60
Fasting Glucose (mg/dl)	87.3 ± 8.9	86.1 ± 9.0	1.2	does not apply	87.2 ± 9.1	86.1 ± 9.0	1.1	does not apply
Fasting Insulin (uUI/dl)	12.0 (9.1)^***^	6.7 (4.9)	5.3	0.77^‡^	13.2 (10.1) ^***^	7.0 (6.4)	5.2	0.81^‡^
HOMA-IR	2.6 (2.3)^***^	1.4 (1.2)	1.2	0.68^‡^	3.0 (2.4) ^***^	1.5 (1.3)	1.5	0.88^‡^
HOMA-S (%)	38.5 (40.3) ^***^	71.4 (63.4)	-32.9	0.60^‡^	33.3 (34.4) ^***^	66.6 (65.6)	33.3	0.57^‡^
HOMA-β (%)	194 (157) ^***^	107 (132)	87	0.61^‡^	207 (155) ^***^	116 (141)	91	0.63^‡^
Disposition Index	0.72 (0.4)	0.75 (0.4)	0.03	does not apply	0.72 (0.3)	0.75 (0.3)	0.03	does not apply
Total Cholesterol (mg/dl)	176.1 ± 34.1^*^	168.6 ± 30.5	7.5	0.24	178.0 ± 32.2^**^	167.5 ± 31.9	10.5	0.33
HDL-cholesterol (mg/dl)	42.1 ± 9.1^***^	51.1 ± 9.7	-9.0	0.95	41.6 ± 10.6^***^	50.1 ± 10.6	-8.5	0.80
LDL-cholesterol (mg/dl)	108.1 ± 29.1	102.7 ± 29.3	5.4	does not apply	109.5 ± 27.2	103.1 ± 29.1	6.4	does not apply
TG-HDL ratio	4.1 (1.4) ^***^	3.3 (1.1)	0.8	0.65^‡^	4.3 (1.2)^***^	3.3 (1.0)	1.0	0.95^‡^
Triglycerides (mg/dl)	118.0 (80.1)^***^	73.6 (42.9)	44.4	0.73	135.7 ± 82.6^***^	77.0 ± 47.5	66.2	0.99

Values expressed as mean ± SD and median (IQR). Two-tailed Student’s t test for independent samples. Wilcoxson Rank-sum test. *Significant at P<0.05. ** Significant at P<0.01. *** Significant at P<0.001. ^†^Cohen’s d statistic (normal distributions), except as indicated. ^‡^Cliff’s δ (non-normal distributions). Values of d of 0.20, 0.50 and 0.80 denote small, medium and large differences. Values of δ are considered small around 0.15, medium around 0.33, and large around 0.47.

**Table 5:** Cardiometabolic profile in pubertal obese patients according to optimal cutoff values of SPISE to predict IR (5.4) and MetS (5.4).

	Insulin Resistance & Metabolic Syndrome			
Pubertal patients (n=293)	SPISE ≤ 5.4 (n=173)	SPISE > 5.4 (n=259)	Mean difference	ES for difference
Waist circumference (mm Hg)	102.1 ± 10.6^***^	86.3 ± 7.8	15.1	1.75
Systolic Blood Pressure (mm Hg)	119 ± 14^***^	110 ± 12	9.0	0.70
Diastolic Blood Pressure (mm Hg)	74± 9^***^	69 ± 8	5.0	0.59
Fasting Glucose (mg/dl)	87.6 ± 8.6	84.8 ± 7.5	1.5	(…)
Fasting Insulin (uUI/dl)	16.6 (9.2)^***^	12.0 (8.1)	4.6	0.54^‡^
HOMA-IR	3.7 (2.3)^***^	2.4(1.9)	1.3	0.63^‡^
HOMA-S (%)	27.4 (17.4) ^***^	41.6 (31.0)	-14.2	0.54^‡^
HOMA-β (%)	275 (272) ^**^	206 (209)	69	0.29^‡^
Disposition Index	0.74 (0.5)	0.76 (0.6)	0.02	(…)
Total Cholesterol (mg/dl)	173.4 ± 35.4^**^	161.6 ± 33.0	11.8	0.35
HDL-cholesterol (mg/dl)	41.3 ± 8.4^***^	44.7 ± 9.4	-5.9	0.38
LDL-cholesterol (mg/dl)	103.1 ± 30.1	96.4 ± 28.3	7.0	(…)
TG-HDL ratio	4.2 (1.5) ^***^	3.4 (1.2)	0.7	0.60^‡^
Triglycerides (mg/dl)	131.9 (78.1)^***^	75.0 (52.2)	56.9	0.89^‡^

Values expressed as mean ± SD and median (IQR). Two-tailed Student’s t test for independent samples. Wilcoxson Rank-sum test. *Significant at P<0.05. ** Significant at P<0.01. *** Significant at P<0.001. ^†^Cohen’s d statistic (normal distributions), except as indicated. ^‡^Cliff’s δ (non-normal distributions). Values of d of 0.20, 0.50 and 0.80 denote small, medium and large differences. Values of δ are considered small around 0.15, medium around 0.33, and large around 0.47.

## Discussion

### Main findings

In this sample of Chilean children and adolescents with obesity attending a weight-control clinical program, we found a high prevalence of IR and MetS. Based on AUC values, in both groups SPISE showed good performance in screening MetS IR. Studies conducted in Hispanic and Caucasian adolescents also found that SPISE had good predictive ability in detecting individuals with high obesity-related cardiometabolic risk. In adolescents from the Santiago Longitudinal Study (n=678, mean age 16.8y, 50% females), SPISE outperformed the TG | HDL ratio and HOMA-IR in the prediction of MetS and IR, with better predictive capacity in males than females (25). In a subset of the Beta-JUDO study (n=99; 13.1y, 41% females), SPISE outperformed HOMA-IR and the Hepatic Insulin Resistance Index (HIRI) in predicting non-alcoholic fatty liver disease (NAFLD). Yet, the ability of SPISE in the prediction of NAFLD was moderate-to-good, in both males (AUC=0.71) and females (AUC=0.74) (26).

Our results suggest no differences in the performance of SPISE to screen both MetS and IR after controlling for the biological development of the patients. Because puberty is associated with a marked decrease in insulin sensitivity, also in healthy youth (28), it seems important to determine whether puberty might interfere with the predictive ability of SPISE. Previous studies did not address this matter, despite including pre-pubertal and pubertal patients (22, 26). After comparing the AUC using the DeLongi test, our results show that the performance of SPISE in the prediction of MetS and IR does not vary according to the Tanner stage of participants. Therefore, SPISE could be used in the pediatric population to identify individuals at risk of obesity-related cardiometabolic alterations.

Interestingly, our results also show that the same SPISE cutoff point (5.4) can be used to predict MetS and IR in adolescents with obesity, which could make straightforward and effective screening procedures. Because people of Hispanic descent are more susceptible to obesity and related health consequences compared to individuals from different ethnic backgrounds (39–41), it is worth testing whether this cutoff would work in other populations and whether a single cutoff would allow screening of both MetS and IR. It is well known that adults with MetS and IR are at increased risk of type-2 diabetes and cardiovascular disease (42, 43). While more evidence on the relationship of MetS and IR in childhood and adolescence with the development of adult cardiometabolic risk is still in need, SPISE could be helpful in early screening subjects at risk, helping focus pediatric care teams on preventive strategies. Furthermore, because SPISE comprises BMI and routine lipids, such as TG and HDL-chol, screening could be conducted in large clinical and epidemiological settings, where those measurements are often available. This is most needed in countries where obesity has reached pandemic proportions also in younger age groups. The latest report on childhood obesity in Chile show that, despite persistent efforts by governments and authorities to mitigate unhealthy eating, obesity rose from 19.5% to 31% in 2009-2021 in children. Over the same period, obesity jumped from 8.7% to 17% in adolescents (8). Overall, 58% of Chilean children and adolescents have either overweight or obesity.

It is worth mentioning that individuals diagnosed with MetS and IR with SPISE also had significantly higher insulin, HOMA-β, and HOMA-S values than children and adolescents not having MetS and IR, according to SPISE. Although SPISE does not include either insulin or glucose in its algorithm, our findings suggest that this surrogate marker of IR could serve to approach β-cell function. In so doing, SPISE could identify subjects at risk of impaired β-cell functioning. Likewise, SPISE could be used to test the effectiveness of lifestyle interventions (e.g., weight loss *via* diet or exercise) designed to enhance β-cell functioning at a lower cost and less demanding logistics compared to insulin-based biomarkers. Hence, future studies should further explore the relationship of SPISE with β-cell function. Since β-cell regenerative capacity is limited in humans, reducing β-cell workload appears to be the most effective way to preserve β-cell functional mass to date, underpinning the importance of lifestyle modification and weight loss for the treatment and prevention of T2D (44, 45). To the best our knowledge, this is the first study connecting SPISE with β-cell functioning.

### Major implications

In the pediatric population, the SPISE algorithm might be a useful tool for estimating IR-related cardiometabolic risk in clinical and large epidemiological settings. Although IR regularly occur in children and adolescents with obesity and relates to a higher risk of major cardiometabolic disorders (13, 46), the prevalence of IR in these groups is still undetermined (19). Diagnostic based on insulin measurements have been unable to provide accurate, reliable, reproducible, and easily applicable measurement procedures in large groups (19). Also in clinical practice, fasting insulin is not advised to determine insulin sensitivity in children (19, 46, 47). Hence, there is a need for screening programs to assess insulin sensitivity without a need to measure insulin. SPISE uses TG, HDL-Chol and BMI, which are more reliable and cheaper to obtain compared to insulin. Also, SPISE requires a single blood draw. Third, this new surrogate of IR brings the possibility to identify children at risk of IR-related cardiometabolic disorders in large groups to support focused preventative interventions. Because of the great number of children and adolescents having or at risk of having obesity and because IR may occur as part of the physiological changes in puberty (28, 29), early detection of impaired insulin sensitivity in pubertal children is pivotal. Population-based health surveys usually have measurements of body composition and lipid profile and, hence, this data could shed light on the magnitude of IR prevalence in the pediatric population.

### Limitations and strengths

This study has limitations worth reckoning. Because our sample was comprised of children and adolescents attending an obesity clinic, our findings cannot be generalized to the overall population of Chilean children and adolescents with obesity, as this is a condition not always receiving medical surveillance. However, it could be extrapolated to children and adolescents with obesity under medical surveillance. Secondly, we used the Cook criteria for MetS diagnosis, although other MetS definitions have been proposed in the pediatric population (48, 49). Yet, the Cook criteria is recommended in clinical practice, since this standard provides a wider diagnostic range compared to other MetS definitions, thereby reducing the risk of underdiagnosis. Similarly, we identify children and adolescents with IR with the HOMA-IR instead of the glucose clamp; this may limit the clinical usefulness of the findings as HOMA-IR is not a formal diagnostic test. While the glucose clamp is the ‘gold standard’ for assessing the insulin action on glucose utilization in humans and animals, it is highly invasive not to mention expensive and time consuming. As a result, it is not recommended in healthy individuals and its use is constrained in routine clinical settings or large epidemiological samples. Because our patients did not suffer from T2D -although they did show risk factors for the disease-, and a very small fraction had impaired fasting glucose (5.1% with no sex or pubertal development-related differences), we chose to work with a less invasive surrogate technique. For the purpose of this study, it is worth noting that Paulmichl et al. found that the screening performance of SPISE (as measured by the area under ROC) was similar to that of the M-value from the glucose clamp and similar to that of HOMA-IR (22), which suggest that HOMA-IR performance in risk assessment of IR might not differ from the performance displayed by the M-value. A third important limitation is the cross-sectional nature of the study, which limits the ability of our findings to conclude on the temporality of these associations. Future studies should longitudinally explore the SPISE ability in predicting the risk of cardiometabolic diseases in the future in obese children and adolescents (50, 51) or subsequent stages of life such as the old age (24). Because these studies were conducted in Caucasian and Asian populations, it would be very valuable to explore this use of SPISE in subjects of Hispanic background. Despite these limitations, our study has several strengths. The prevalence rate of obesity and cardiometabolic risk is high in children and adolescents from Chile, where 58% of the pediatric population suffers from some degree of excess weight, according to the latest national reports (8). Also, our results provide further evidence of a relatively new biomarker that allows good early screening of children and adolescents with IR-related cardiometabolic risk, using an easy-to-estimate, low-cost indicator, which might be potentially helpful in both clinical and population settings. Our results suggest no differences in the effectiveness of SPISE to identify adolescents at greater cardiometabolic risk, after controlling for pubertal status. This has not been described in previous validity assessments of the SPISE. Last, our participants are of Hispanic descents, thus, they belong to an ethnic group with greater susceptibility to impaired insulin sensitivity and cardiometabolic alterations (40, 41).

## Data availability statement

The raw data supporting the conclusions of this article will be made available by the authors, without undue reservation.

## Ethics statement

The studies involving human participants were reviewed and approved by Comité Ético-Científico, Instituto de Nutrición y Tecnología de los Alimentos, Universidad de Chile. Written informed consent to participate in this study was provided by the participants’ legal guardian/next of kin.

## Author contributions

Conceptualization, RB, PC. Methodology, RB, PC. Formal analysis, PC, MM. Investigation, RB, PC, MM. Data curation, PC, VT, DZ. Writing—original draft preparation, PC. Writing—review and editing, VT, DZ, MA. Supervision, RB, VT, MA. Project administration, RB, PC. Funding acquisition, RB. All authors contributed to the article and approved the submitted version.
